# Desempenho Diagnóstico da Angiotomografia Computadorizada e da Avaliação Seriada de Troponina Cardíaca Sensível em Pacientes com Dor Torácica e Risco Intermediário para Eventos Cardiovasculares

**DOI:** 10.36660/abc.20210006

**Published:** 2022-05-04

**Authors:** Alexandre de Matos Soeiro, Bruno Biselli, Tatiana C.A.T. Leal, Aline Siqueira Bossa, Maria Cristina César, Sérgio Jallad, Priscila Gherardi Goldstein, Patrícia Oliveira Guimarães, Carlos Vicente Serrano, Cesar Higa Nomura, Débora Nakamura, Carlos Eduardo Rochitte, Paulo Rogério Soares, Múcio Tavares de Oliveira

**Affiliations:** 1 Instituto do Coração Faculdade de Medicina Universidade de São Paulo São Paulo SP Brasil Instituto do Coração (InCor) - Faculdade de Medicina da Universidade de São Paulo, São Paulo, SP – Brasil

**Keywords:** Doenças Cardiovasculares, Fatores de Risco, Controle de Riscos, Dor no Peito, Tomografia Computadorizada por Imagem Raios X/métodos, Troponina T, Troponina I, Angiotomografia Coronária/métodos

## Abstract

**Fundamento:**

A angiotomografia coronária (ATC) tem sido usada para avaliação de dor torácica principalmente em pacientes de baixo risco, e poucos dados existem com pacientes em risco intermediário.

**Objetivo:**

Avaliar o desempenho de medidas seriadas de troponinas sensíveis e de ATC em pacientes de risco intermediário.

**Métodos:**

Um total de 100 pacientes com dor torácica, TIMI score 3 ou 4 e troponina negativa foram prospectivamente incluídos. Todos os pacientes foram submetidos à ATC, e aqueles com obstruções ≥ 50% foram encaminhados à cineangiocoronariografia. Pacientes com lesões < 50% recebiam alta hospitalar, receberam alta e foram contatados 30 dias depois por telefonema para avaliação dos desfechos clínicos. Os desfechos foram hospitalização, morte, e infarto agudo do miocárdio em 30 dias. A comparação entre os métodos foi realizada pelo teste de concordância kappa. O desempenho das medidas de troponina e da ATC na detecção de lesões coronárias significativas e desfechos clínicos foi calculado. Os resultados foram considerados estatisticamente significativos quando p <0,05.

**Resultados:**

Estenose coronária ≥ 50% na ATC foi encontrada em 38% dos pacientes e lesões coronárias significativas na angiografia coronária foram encontradas em 31 pacientes. Dois eventos clínicos foram observados. A análise de concordância Kappa mostrou baixa concordância entre as medidas de troponina e ATC na detecção de lesões coronárias significativas (kappa = 0,022, p = 0,78). O desempenho da ATC para detectar lesões coronárias significativas na angiografia coronária ou para prever eventos clínicos em 30 dias foi melhor que as medidas de troponina sensível (acurácia de 91% versus 60%).

**Conclusão:**

ATC teve melhor desempenho que as medidas seriadas de troponina na detecção de doença coronariana significativa em pacientes com dor torácica e risco intermediário para eventos cardiovasculares.

## Introdução

Dor torácica é uma das principais queixas nos serviços de emergências em todo o mundo. Grandes avanços foram feitos na prática clínica com o uso da angiotomografia coronária (ATC) e de troponina de alta sensibilidade no diagnóstico de síndrome coronária aguda (SCA).^1 - 4^

Os ensaios para detecção das troponinas T e I sensíveis e ultrassensíveis possuem limiares de detecção para lesão miocárdica 10 a 100 vezes menores que troponinas convencionais. Esses métodos têm melhor acurácia para o diagnóstico da SCA particularmente em pacientes com dor torácica de curta duração. A maioria dos estudos avaliando a acurácia de medidas repetidas de troponina em excluir SCA incluiu paciente com baixo risco de eventos cardiovasculares, como avaliado pelos escores de risco TIMI (trombólise no infarto do miocárdio), HEART e GRACE.^5 , 6^

A avaliação anatômica da árvore coronária usando ATC tem um papel importante na exclusão de SCA em pacientes com risco baixo a intermediário para doença arterial coronariana. Achados de ATC se correlacionaram bem com angiografia coronária invasiva em um estudo que incluiu 230 pacientes com dor torácica. Observaram-se alta sensibilidade e especificidade, e valores preditivos negativos quando as lesões eram maiores que 50% na ATC. No estudo ROMICAT-II, a estratégia com ATC foi tão segura como a estratégia convencional em termos de ocorrência de eventos cardiovasculares maiores em 28 dias. Assim, a ATC é um método não invasivo preciso para detecção de SCA em pacientes com dor torácica aguda. No entanto, sua validação foi feita principalmente em pacientes com perfis de baixo risco.^7^ O presente estudo teve como objetivo avaliar o desempenho da medida de troponina sensível e da ATC na detecção de lesões coronárias significativas na angiografia coronária e eventos clínicos em pacientes com dor torácica e risco intermediário para eventos cardiovasculares.

## Métodos

### Pacientes

**Figura 1:**
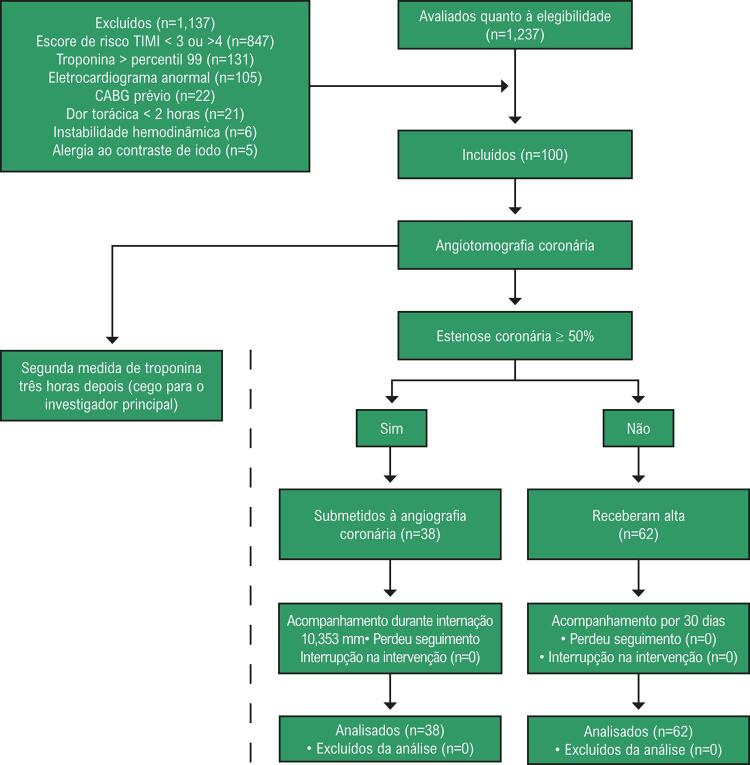
– Delineamento e fluxograma do estudo; CABG: coronary artery bypass grafting (revascularização com enxerto de bypass da artéria coronária).

O delineamento do estudo está apresentado na Figura 1 . Nós incluímos prospectivamente um total de 100 pacientes com dor torácica na admissão no serviço de emergência do Instituto do Coração (InCor) da Faculdade de Medicina da Universidade de São Paulo, Brasil. Foram incluídos pacientes com idade entre 40 e 75 anos, apresentando dor torácica com duração de pelo menos duas horas antes da chegada ao serviço, com um risco TIMI de 3 ou 4. Além disso, para a inclusão no estudo, um novo (ou provavelmente novo) desvio de ST de no mínimo 0,5 mV e/ou inversão de onda T de pelo menos 0,2 mV não deviam estar presentes no eletrocardiograma, e a primeira medida da troponina sensível deveria ser < percentil 99. Os critérios de exclusão foram gestantes, pacientes com instabilidade hemodinâmica, creatinina sérica > 1,5 mg/dL, intolerância a betabloqueadores, alergia a contraste com iodo, asma, trauma torácico nos últimos 30 dias, índice de massa corporal > 40 Kg/m^2^_,_ história de revascularização (bypass) da artéria coronária, e lesão da artéria coronária ≥ 50%. O estudo foi aprovado pelo comitê de ética para estudos envolvendo seres humanos de nossa instituição, e todos os participantes assinaram o termo de consentimento. Não há conflito de interesse de nenhum autor.

### Troponina sensível

As amostras de sangue foram colhidas para a determinação de troponina I sensível em dois momentos: na admissão e três horas depois. Os investigadores desconheciam (cegos) essa segunda medida até o final do estudo. A determinação quantitativa de troponina I foi obtida por um imunoensaio tipo sanduíche realizado em três estágios, que utiliza a tecnologia de quimioluminescência direta e quantidades constantes de dois anticorpos monoclonais. Um reagente foi adicionado para detectar as ligações não específicas. Foi usado um kit comercial ADVIA Centaur^®^ TnI-Ultra (Siemens Healthcare Diagnostics, Tarrytown, NY, EUA) em um equipamento automático do mesmo fabricante.

### Angiotomografia coronária

Após a inclusão no estudo, todos os pacientes foram submetidos à ATC. As imagens da ATC foram adquitidas usando um tomógrafo de 320 detectores (Aquilion ONE, Canon Medical Systems, Japão) e um protocolo padrão de escaneamento. Para atingir uma frequência cardíaca menor que 65 bpm durante a aquisição, os pacientes receberam metoprolol via oral (50-100 mg).

### Desfechos do estudo

Dois subgrupos foram estudados: a) pacientes com estenose coronária ≥ 50%; na ATC e que se submeteram à angiografia coronária, e b) pacientes cuja ATC não mostrou nenhuma lesão ou apresentou lesões < 50%, que receberam alta e foram contatados 30 dias depois por telefonema para avaliação dos desfechos clínicos. Lesões coronárias ≥ 70% na angiografia coronária foram consideradas significativas. Desfechos clínicos de interesse foram internação hospitalar, morte, e infarto do miocárdio.

### Análise estatística

Os dados foram analisados usando o programa SAS Statview 5.0. Análise descritiva das características basais foi realizada usando médias e desvios padrões quando se assumiu distribuição normal dos dados, e medianas e intervalos interquartis foram usados para dados sem distribuição normal. O teste de Kolmogorov-Smirnov foi usado para avaliar a normalidade da distribuição das variáveis contínuas. Comparações do tempo entre a chegada do paciente até a segunda medida de troponina e o tempo entre a chegada até ATC foram feitas pelo teste t não pareado.

Comparação entre os métodos diagnósticos foi realizada pelo teste de concordância Kappa. Nós comparamos a concordância entre as medidas de troponina e lesões coronárias significativas detectadas na angiografia coronária ou desfechos clínicos. Os resultados foram considerados estatisticamente significativos quando p<0,05. Calculamos a sensibilidade, a especificidade, valores preditivos positivos, valores preditivos negativos e acurácia da troponina sensível na detecção de lesões coronárias significativas no subgrupo que se submeteu à angiografia coronária (n=38). O desempenho foi calculado usando como resultado positivo uma concentração de troponina acima do percentil 99 na segunda medida, e a porcentagem de variação do método em relação à primeira medida, identificando-se o melhor ponto de corte pela curva ROC. Foi realizada uma análise complementar calculando-se a área sob a curva ROC e o escore de corte do aumento (%) nos níveis de troponina e lesões coronárias significativas detectadas por angiografia coronária e eventos clínicos ou somente por angiografia coronária.

Com base em um erro alfa de 0,05 e usando um poder de 0,8 para desfechos primários, nós calculamos que o número de indivíduos necessários para este estudo seria de 71 ou mais de acordo com estudos prévios, considerando a incidência de lesões coronárias com estenose ≥ 50% em risco intermediário (TIMI), uso de ATC em cerca de 24% dos pacientes,^8^ e diagnóstico de SCA pelo uso de troponina sensível em 11,4% dos pacientes submetidos a protocolos de dor torácica.^6^ Esses dados foram usados para avaliar a hipótese de uma diferença no desempenho entre esses métodos.

## Resultados

### População do estudo

Foram incluídos 100 pacientes consecutivos com dor torácica aguda e escores de risco TIMI de 3 ou 4. Características clínicas dos pacientes estão apresentadas na Tabela 1 . A inclusão e o acompanhamento dos pacientes foram realizados entre abril de 2016 e março de 2019 quando o tamanho amostral estimado previamente foi alcançado. A idade média dos pacientes foi 62,9 ± 10,5 anos e 58% eram do sexo feminino. A maioria da população (81%) do estudo tinha um risco TIMI de 3. Variações de 20% na troponina sensível foram observadas em 29 pacientes. Estenose coronária ≥ 50% na ATC foi observada em 38% dos pacientes (≥ 70% em 25 pacientes). Todos esses foram submetidos à angiografia coronária. Lesões coronárias significativas na angiografia coronária foram observadas em 31 pacientes.

**Tabela 1:** – Características basais dos pacientes do estudo (n=100)

Características demográficas	
Idade (anos)	62,9 (± 10,5)
Sexo masculino	42 (42%)
**Comorbididades/fatores de risco**	
Hipertensão	87 (87%)
Diabetes	49 (49%)
Dislipidemia	79 (79%)
AVC /AIT prévio	5 (5%)
IAM prévio	18 (18%)
ICP prévia	16 (16%)
Tabagismo (atual ou prévio)	52 (52%)
História familiar de DAC	44 (44%)
**Apresentação clínica**	
Pressão arterial sistólica (mmHg)	144,9 (± 23,7)
Frequência cardíaca (bpm)	71,9 (± 13,0)
Escore de risco TIMI de 3	81 (81%)
Escore de risco TIMI de 4	19 (19%)
**Resultados laboratoriais**	
Hemoglobina (g/dL)	13,8 (± 1,5)
Creatinina (mg/dL)	0,92 (± 0,3)

*AVC: acidente vascular cerebral; AIT: ataque isquêmico transitório, IAM: infarto agudo do miocárdio; DAC: doença arterial coronariana; ICP: intervenção coronária percutânea; TIMI: trombólise no infarto do miocárdio.*

### Eventos clínicos

Todos os pacientes estavam vivos no dia 30. Entre os pacientes que receberam alta sem angiografia coronária, foram observadas duas novas internações em 30 dias. Não houve ocorrência de morte ou infarto do miocárdio durante o acompanhamento.

### Análise de concordância na população geral

#### Concordância entre troponina sensível e achados na ATC

Esta análise incluiu todos os pacientes do estudo (n=100). O teste de concordância kappa revelou uma leve concordância entre a segunda medida de troponina positiva e ATC na detecção de lesões coronárias significativas (kappa = 0,022; p = 0,78). O tempo decorrido entre a chegada do paciente e a segunda medida de troponina foi 312,08 ± 82,39 minutos versus 256,70 ± 83,91 minutos entre a chegada do paciente e a ATC (p < 0,0001). O tempo médio entre a ATC até a alta foi 6837,10 ± 8068,17 minutos, e todos os pacientes foram submetidos à angiografia coronária nas primeiras 24 horas de admissão. Cinco pacientes mostram um resultado positivo de troponina na segunda medida, mas receberam alta do hospital uma vez que não apresentaram lesões significativas na ATC.

#### Concordância entre variações na troponina sensível e presença de lesões coronárias significativas na angiografia coronária ou ocorrência de eventos clínicos

**Figura 2:**
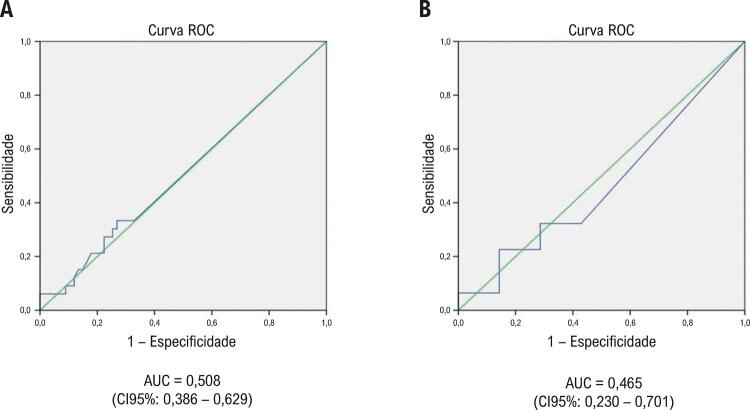
– Curva ROC para a variação de 20% na troponina sensível e detecção de (A) lesões significativas na angiografia coronária ou ocorrência de eventos e (B) lesões significativas somente por angiografia coronária. AUC: área sob a curva; IC: intervalo de confiança.

Usando a presença de lesões coronárias significativas na angiografia coronária ou a ocorrência de eventos clínicos em 30 dias como o padrão ouro, o teste de concordância de kappa com a segunda medida de troponina positiva mostrou uma leve concordância (kappa = 0,002, p=0,979). O melhor ponto de corte para a variação de troponina do momento basal até a segunda medida quanto à presença de lesões coronárias significativas na angiografia coronária ou ocorrência de eventos clínicos em 30 dias foi de 20%. A área sob a curva ROC para a variação de 20% na troponina sensível foi 0,508 (IC95% 0,386 – 0,629) ( Figura 2 ).

#### Desempenho das medidas de troponina sensível ou lesões coronárias ≥ 50% na ATC na detecção de lesões coronárias importantes na angiografia coronária ou ocorrência de eventos clínicos

A sensibilidade, a especificidade, valor preditivo positivo, valor preditivo negativo, e acurácia: a) da segunda medida de troponina positiva; b) de variações de 20% nos níveis de troponina; e c) de lesões coronárias ≥ 50% na ATC para detecção de lesões ≥70% na angiografia coronária ou para predição de eventos clínicos em 30 dias estão apresentados na Tabela 2 . O desempenho geral da ATC para detectar os desfechos compostos foi melhor que o as concentrações de troponina.

**Tabela 2:** – Desempenho da segunda medida de troponina positiva, variação de troponina de 20%, e lesões coronárias ≥ 50% na angiotomografia coronariana na detecção de lesões ≥ 70% na angiografia coronária ou na predição de eventos clínicos em 30 dias

	Sensibilidade	Especificidade	Valor preditivo positivo	Valor preditivo negativo	Acurácia
Segundo resultado positivo de troponina	12,1%	88,1%%	33,3%	67,0%	63,0%
Variação na troponina ≥ 20%	33,3%	73,1%	37,9%	69,0%	60,0%
Lesões coronárias ≥ 50% na ATC	93,9%	89,6%	81,6%	96,8%	91,0%

*ATC: angiotomografia computadorizada.*

## Análise de concordância em pacientes que se submeteram à angiografia coronária

### Concordância entre variações nas medidas de troponina e presença de lesões coronárias significativas na angiografia coronária

Esta análise incluiu somente os pacientes que se submeteram à angiografia coronária (n=38). Usando a presença de lesões coronárias significativas na angiografia coronária como padrão ouro, o teste de concordância kappa, com um resultado de troponina positivo na segunda medida, mostrou concordância leve (kappa = 0,006; p=0,922). O melhor ponto de corte para a variação de troponina quanto à presença de lesões coronárias significativas na angiografia coronária foi 20%. A área sob a curva ROC para a variação de 20% na troponina sensível foi 0,465 (IC95% 0,230 – 0,701) ( Figura 2 ).

### Desempenho das medidas de troponina sensível na detecção de lesões coronárias significativas na angiografia coronária

A sensibilidade, a especificidade, valor preditivo positivo, valor preditivo negativo, e acurácia: a) da segunda medida de troponina positiva; e b) de variações de 20% nos níveis de troponina na detecção de lesões ≥70% na angiografia coronária estão apresentados na Tabela 3 . Lesões coronárias importantes foram detectadas por angiografia coronária em 81,6% dos pacientes com lesões ≥ 50% na ATC. Essa proporção foi maior em comparação à troponina positiva na segunda medida ou suas variações. Elevada especificidade foi encontrada para resultado de troponina positivo na segunda medida, e variações de troponina de 20% - 85,7% e 71,4%, respectivamente.

**Tabela 3:** – Desempenho do resultado positivo de troponina na segunda medida e da variação de troponina de 20% na detecção de lesões ≥ 70% na angiografia coronária

	Sensibilidade	Especificidade	Valor preditivo positivo	Valor preditivo negativo	Acurácia
Segundo resultado positivo de troponina	12,9%	85,7%%	80%	18,2%	26,3%
Variação na troponina ≥ 20%	32,3%	71,4%	83,3%	19,2%	39,5%

## Discussão

Nosso estudo apresenta o desempenho de medidas de troponina sensível ou da ATC na detecção de lesões coronárias significativas na angiografia coronária e/ou de eventos clínicos em pacientes com dor torácica e risco intermediário para eventos cardiovasculares. As medidas de troponina sensível mostraram fraca concordância com a detecção de lesões coronárias significativas na ATC o uma angiografia coronária, e com a ocorrência de eventos clínicos. A ATC foi superior à medida de troponina seriada, com melhor sensibilidade e valor preditivo negativo na detecção de doença arterial coronariana. Na prática clínica, os pacientes com dor torácica aguda normalmente recebem alta hospitalar de acordo com protolocos de dor torácica baseados somente na medida seriada de troponina. Nossos achados sugerem que pacientes em risco intermediário sem alterações isquêmicas no eletrocardiograma deveriam ser estratificados na admissão por ATC. Enfatizamos o fato de que a avaliação de eventos clínicos é secundária nesse contexto devido ao tamanho amostral e, assim, a concordância com o diagnóstico de doença coronariana foi nosso principal achado.

Não é incomum que pacientes com dor torácica sejam liberados das salas de emergências após avaliação inicial, e desenvolvam eventos isquêmicos nas horas seguintes. Esses indivíduos não recebem tratamento adequado em tempo apropriado,^3 , 9^ e estima-se que um em cada oito pacientes com angina instável sofre um infarto agudo do miocárdio (IAM) nas duas semanas seguintes. A mortalidade em pacientes com IAM admitidos ou erroneamente liberados dos departamentos de emergência varia entre 6% e 25%,^3^ o que leva a ações judiciais relacionadas a mal práticas.^4^ A incidência de eventos adversos em pacientes com escore de risco TIMI de 3 e 4 pode ser de até 11,1%.^10 , 11^ No entanto, estudos sobre estratégias diagnósticas para avaliação de dor torácica nessa população específica são escassos e derivam principalmente de análises secundárias.

Em 2006, Morris et al.,^8^ conduziram um estudo incluindo 1000 pacientes consecutivos com dor torácica aguda para investigar se o uso do escore de risco TIMI ajuda a predizer eventos combinados em 30 dias nessa população. A AUC foi 0,79 (IC 95%: 0,75 – 0,84), que mostrou uma boa aplicabilidade do escore. Desde então, estudos sugerem que pacientes com escore intermediário (3 e 4) deveriam ter os níveis de troponina medidos e submetidos a um teste isquêmico provocativo sempre que possível.^8 , 12^ Nossos resultados também sugerem que é uma melhor avaliação da presença de doença coronariana por meio de teste não invasivo além das medidas de troponina nessa população.

A realização de duas medidas seriadas de troponina sensível com intervalos curtos de tempo é uma boa abordagem para excluir SCAs em pacientes em baixo risco, o que permite a implementação de protocolos de avaliação rápida de dor torácica.^5 , 6 , 13 , 14 - 31^ Um estudo com pacientes com suspeita de SCA mostrou que um aumento de 20% nos níveis de troponina ultrassensível foi associado com maior probabilidade de SCA, com uma área sob a curva ROC de 0,785. Outros estudos relataram que variações na troponina T ultrassensível ao longo de algumas horas mostraram valores preditivos negativos mais altos.^32^ Contudo, vale mencionar que a população incluída na maioria dos estudos que mostraram uma acurácia muito alta da troponina ultrassensível era de baixo risco. Em nosso estudo, o valor preditivo negativo de uma variação de 20% na troponina sensível foi de 69%, um índice abaixo do apontado na literatura, o que pode ser justificado pela inclusão de pacientes em risco intermediário.

No estudo ROMICAT, o uso de ATC além do escore de risco TIMI aumentou a acurácia para predição de eventos.^33^ O estudo ROMICAT-II incluiu 1000 pacientes com dor torácica e primeiro resultado negativo de troponina, que foram randomizados para serem submetidos à ATC ou seguir o protocolo usual de dor torácica.^34^ Durante os dois anos de acompanhamento incluindo 333 pacientes, observou-se que a ATC apresentou poder preditivo elevado, com uma área sob a curva de 0,61 para eventos cardiovasculares combinados. Quando associada ao escore TIMI, a AUC alcançou 0,84. Nesse estudo, somente 5,4% dos pacientes apresentaram um escore TIMI entre 3 e 4.^35 , 36^ Nós acreditamos que nossos achados contribuem para esses resultados, uma vez que, em pacientes em risco intermediário, a ATC teve um desempenho melhor que as medidas seriadas de troponina sensível.

Em comparação aos protocolos de dor torácica tradicionais, a ATC não altera desfechos tais como morte ou IAM, embora reduza o período de internação e o número de internações desnecessárias.^5 , 7 , 37 - 56^ Litt et al.,^7^ mostraram que o uso de ATC, em comparação ao protocolo tradicional de avaliação de dor torácica, apresentou um excelente perfil de segurança em pacientes de baixo risco, pois não houve morte ou IAM em 30 dias. Ainda, a ATC possibilitou um maior número de altas hospitalares (49,6% vs. 22,7%) e menor permanência hospitalar (18 horas vs. 24,8 horas, p < 0,001). A concordância entre a ATC e os achados do cateterismo cardíaco também parece ser alta.^44 , 45^ No estudo ROMICAT-II, o tempo de internação foi 7,6 horas menor no grupo ATC em comparação ao grupo de cuidado usual. Em nosso estudo, nós observamos que o intervalo de tempo entre a chegada do paciente e a ATC foi aproximadamente uma hora menor que o intervalo entre a chegada do paciente e o resultado da segunda medida de troponina.

Algumas limitações devem ser consideradas na interpretação de nossos resultados. Este foi um estudo unicêntrico, com um tamanho amostral relativamente pequeno, e o número de eventos clínicos foi baixo. Assim, estudos maiores devem ser realizados para validar nossos achados. Pacientes com risco intermediário foram raramente representados em estudos anteriores investigando estratégias de avaliação de dor torácica. Nossos resultados sugerem que a ATC tem um papel importante na exclusão de SCAs nessa população, e acreditamos que esses achados contribuam para a literatura existente.

## Conclusões

A ATC apresentou melhor desempenho que as medidas de troponina sensível na detecção de doença coronariana importante em pacientes com dor torácica e risco intermediário para eventos cardiovasculares.
