# Supplementary material for: Desempenho Diagnóstico da Angiotomografia Computadorizada e da Avaliação Seriada de Troponina Cardíaca Sensível em Pacientes com Dor Torácica e Risco Intermediário para Eventos Cardiovasculares
Source: Arq Bras Cardiol. 2022 May 4;118(5):894–902. [Article in Portuguese] doi: 10.36660/abc.20210006 (PMC9368885; doi:10.36660/abc.20210006)
Supplement: Translation

# Translation: Diagnostic Performance of Coronary Tomography Angiography and Serial Measurements of Sensitive Cardiac Troponin in Patients With Chest Pain and Intermediate Risk for Cardiovascular Events

**Authors:** Alexandre de Matos Soeiro, Bruno Biselli, Tatiana C.A.T. Leal, Aline Siqueira Bossa, Maria Cristina César, Sérgio Jallad, Priscila Gherardi Goldstein, Patrícia Oliveira Guimarães, Carlos Vicente Serrano, Cesar Higa Nomura, Débora Nakamura, Carlos Eduardo Rochitte, Paulo Rogério Soares, Múcio Tavares de Oliveira

**Affiliations:** 1 Brazil Instituto do Coração (InCor) - Faculdade de Medicina da Universidade de São Paulo, São Paulo, SP – Brazil

**Keywords:** Cardiovascular Diseases, Risk Factors, Risk Management, Chest Pain, Tomography, X-Ray Computed/methods, Troponin T, Troponin I, Angiotomography Coronary/methods

## Abstract

**Background:**

Coronary tomography angiography (CTA) has been mainly used for chest pain evaluation in low-risk patients, and few data exist regarding patients at intermediate risk.

**Objective:**

To evaluate the performance of serial measures of sensitive troponin and CTA in intermediate-risk patients.

**Methods:**

A total of 100 patients with chest pain, TIMI risk scores of 3 or 4, and negative troponin were prospectively included. All patients underwent CTA and those with coronary stenosis ≥ 50% were referred to invasive coronary angiography. Patients with coronary lesions <50% were discharged and contacted 30 days later by a telephone call to assess clinical outcomes. Outcomes were hospitalization, death, and myocardial infarction at 30 days. The comparison between methods was performed by Kappa agreement test. The performance of troponin measures and CTA for detecting significant coronary lesions and clinical outcomes was calculated. Results were considered statistically significant when p < 0.05.

**Results:**

Coronary stenosis ≥ 50% on CTA was found in 38% of patients and significant coronary lesions on coronary angiography were found in 31 patients. Two clinical events were observed. Kappa agreement analysis showed low agreement between troponin measures and CTA in the detection of significant coronary lesions (kappa = 0.022, p = 0.78). The performance of CTA for detecting significant coronary lesions on coronary angiography or for predicting clinical events at 30 days was better than sensitive troponin measures (accuracy of 91% versus 60%).

**Conclusion:**

CTA performed better than sensitive troponin measures in the detection of significant coronary disease in patients with chest pain and intermediate risk for cardiovascular events.

## Introduction

Chest pain is one of the most common complaints in emergency rooms worldwide. Great advances in clinical practice have been achieved with the use of coronary tomography angiography (CTA) and high-sensitivity troponin in the diagnosis of acute coronary syndrome (ACS).^1 - 4^

Sensitive and high-sensitivity troponin T and I assays have detection thresholds for myocardial injury 10 to 100 times lower than conventional troponins. These assays have better accuracy for the diagnosis of ACS, particularly in patients with short-term chest pain. Most studies evaluating the accuracy of repeated troponin measures in ruling out ACS included patients with low risk for cardiovascular events as assessed by the TIMI (thrombolysis in myocardial infarction), HEART or GRACE risk scores.^5 , 6^

The anatomic evaluation of the coronary tree using CTA has a special role in the exclusion of ACS in patients at low-to-intermediate risk for coronary artery disease. CTA findings correlated well with invasive coronary angiography in a study that included 230 patients with chest pain. High sensitivity and specificity, and negative predictive values were seen when lesions greater than 50% were found in CTA. In the ROMICAT-II trial, the CTA strategy was as safe as the usual care strategy with respect to major cardiovascular events at 28 days. Therefore, CTA is an accurate non-invasive method for detecting ACS in patients with acute chest pain. However, its validation was mainly done in patients with low-risk profiles.^7^ The present study aims to evaluate the performance of sensitive troponin assays and CTA in the detection of significant coronary lesions on coronary angiography and clinical events in patients with chest pain and intermediate risk for cardiovascular events.

## Methods

### Study Patients

The study design is presented in Figure 1 . We prospectively included a total of 100 patients presenting with chest pain at the Emergency Department of the Heart Institute, InCor, University of Sao Paulo Medical School, Sao Paulo, Brazil. To be included, patients had to be aged between 40 and 75 years old, present with chest pain for at least two hours before arrival and have a TIMI risk score of 3 or 4. Additionally, a new or probable new deviation of ST of at least 0.5 mV and/or T wave inversion of at least 0.2 mV should not be present on the electrocardiogram, and their first measure of sensitive troponin should be < 99 percentile for trial inclusion. Exclusion criteria comprised: pregnancy, hemodynamic instability, serum creatinine > 1.5 mg/dL, intolerance to beta-blockers, allergy to iodine contrast, asthma, thoracic trauma in the previous 30 days, body mass index > 40 kg/m2, previous coronary artery bypass graft surgery, and known coronary lesion > 50%. Our Institutional Review Board for human subject studies approved this study, and all participants provided written informed consent prior to enrolment. There is no conflict of interest of any author.

**Figure 1:** – Study design and flowchart; CABG: coronary artery bypass grafting.

### Sensitive troponin assay

Blood samples were drawn for sensitive troponin I measurement at two time points: at presentation and three hours later. The investigators were blinded to the second measurement until the end of the study period. Quantitative determination of troponin I was made by a sandwich-type immunoassay performed in three stages that uses direct chemiluminescence technology and constant quantities of two monoclonal antibodies. A reagent was added for detection of non-specific bindings. The commercial kit ADVIA Centaur^®^TnI-Ultra (Siemens Healthcare Diagnostics, Tarrytown, NY, USA) was used for this in an automated equipment of the same brand. The 99th percentile value was 0.04 ng/ml. Sensitive troponin assays was donated by Siemens Healthcare Diagnostics.

### Coronary tomography angiography

After trial inclusion, all patients underwent CTA. CTA images were acquired using a 320 detector-row scanner (Aquilion ONE, Canon Medical Systems, Japan) and a standard scanning protocol. To reach a heart rate lower than 65 bpm during acquisition, patients received oral metoprolol (50-100 mg).

### Study outcomes

Two subgroups were studied: a) patients who underwent coronary angiography when CTA showed coronary stenosis ≥ 50%; and b) patients whose CTA showed no lesions or lesions < 50% and were discharged and contacted 30 days later by a telephone call to assess clinical outcomes. Coronary lesions > 70% at coronary angiography were considered significant. Clinical outcomes of interest were hospitalization, death and myocardial infarction.

### Statistical analysis

Data were analyzed with the SAS Statview 5.0 software. Descriptive analysis of baseline characteristics was performed using means and standard deviations when a normal distribution of data was assumed, and median and interquartile intervals were used in non-normal distribution. The Kolmogorov-Smirnov test was used to assess the normality of distribution of continuous variables. Comparison of the time from patient arrival to the second troponin test *versus* time from patient arrival to CTA was made using the unpaired T-test.

The comparison between diagnostic methods was performed through Kappa agreement analysis. We compared the agreement between troponin measures and significant coronary lesions on coronary angiography or clinical outcomes. The results were considered statistically significant when *p* < 0.05. We calculated the sensitivity, specificity, positive predictive values, negative predictive values and accuracy of sensitive troponin or CTA in the detection of significant coronary lesions in coronary angiography or clinical events in the whole population (N=100). We also calculated the performance of troponin measures in the detection of significant coronary lesions in the subpopulation who underwent coronary angiography (N=38). Performance was calculated using as positivity a second troponin result above the 99th percentile and the percentage variation of the method in relation to the first measurement, identifying the best cutoff point by the ROC curve. Complementary analysis was made by calculating the area under the ROC curve and the cut-off score of troponin percentage increase and significant coronary lesions determined by coronary angiography and clinical events or by coronary angiography alone.

Based on an alpha error of 0.05 and using a power of 0.8 for primary outcomes, the number of individuals needed for this study was at least 71 according to previous studies, considering the incidence of coronary lesions with stenosis greater than or equal to 50% in patients at intermediate risk (TIMI risk), use of CTA in around 24% of patients,^8^ and diagnosis of ACS by the use of sensitive troponin in 11.4% of patients undergoing chest pain protocols.^6^ These data were used to evaluate the hypothesis of an existing difference in performance between these methods.

## Results

### Study population

A total of 100 patients with acute chest pain and TIMI risk scores of 3 or 4 were consecutively included. Clinical characteristics are presented in Table 1 . Inclusion and follow-up of patients were performed between April 2016 and March 2019 when the previously estimated sample size was reached. Overall, mean age was 62.9 ± 10.5 years and 58% were female. Most of the study population (81%) had a TIMI risk score of 3. Sensitive troponin variations of 20% were observed in 29 patients. Coronary stenosis ≥ 50% on CTA was found in 38% of patients (≥ 70% in 25 patients), and all of them underwent coronary angiography. Significant coronary lesions at coronary angiography were found in 31 patients.

**Table 1:** – Baseline characteristics of study patients (n=100)

Demographic characteristics	
Age (years)	62.9 (± 10.5)
Male sex	42 (42%)
**Comorbidities/risk factors**	
Hypertension	87 (87%)
Diabetes	49 (49%)
Dyslipidemia	79 (79%)
Previous stroke/TIA	5 (5%)
Previous acute MI	18 (18%)
Previous PCI	16 (16%)
Smoking (current or previous)	52 (52%)
Family history of CAD	44 (44%)
**Clinical presentation**	
Systolic blood pressure (mmHg)	144.9 (± 23.7)
Heart rate (bpm)	71.9 (± 13.0)
TIMI risk 3	81 (81%)
TIMI risk 4	19 (19%)
**Laboratory results**	
Hemoglobin (g/dL)	13.8 (± 1.5)
Creatinine (mg/dL)	0.92 (± 0.3)

*CAD: coronary artery disease; MI: myocardial infarction; PCI: percutaneous coronary intervention; SD: standard deviation; TIA: transient ischemic attack; TIMI: thrombolysis in myocardial infarction.*

### Clinical Events

All patients were alive at 30 days. In patients discharged without coronary angiography, two new hospitalizations were observed at 30 days. There were no observed deaths or myocardial infarctions in the follow-up.

### Agreement analysis in the overall population

#### Agreement between sensitive troponin and CTA findings

This analysis included all patients of the study (N=100). The kappa agreement test showed a slight agreement between the second measure of positive troponin and CTA in the detection of significant coronary lesions (kappa = 0.022, p = 0.78). The time between patient arrival and the result of the second troponin was 312.08 ± 82.39 minutes versus 256.70 ± 83.91 minutes between patient arrival and CTA (p < 0.0001). The mean time between CTA to discharge was 6,837.10 ± 8,068.17 minutes, and all patients were submitted to coronary angiography in the first 24 hours of admission. Five patients showed a positive troponin result in the second measurement but were discharged as they did not present significant lesions on CTA.

#### Agreement between sensitive troponin variations and the presence of significant coronary lesions at coronary angiography or the occurrence of clinical events

Using the presence of significant coronary lesions at coronary angiography or the occurrence of clinical events at 30 days as the gold standard, the Kappa agreement test with a positive troponin result in the second measurement showed a slight agreement (kappa = 0.002, p = 0.979). The best cut-off for troponin variation from baseline to the second measure regarding the presence of significant coronary lesions on coronary angiography or the occurrence of clinical events at 30 days was 20%. The area under the ROC curve for the 20% variation in sensitive troponin was 0.508 (CI 95%: 0.386 – 0.629) ( Figure 2 ).

**Figure 2:** – ROC curve for 20% variation in sensitive troponin and detection of (A) significant coronary lesions at coronary angiography and the occurrence of clinical events and (B) significant coronary lesions at coronary angiography only. AUC: area under curve; CI: confidence interval

#### Performance of sensitive troponin measures or coronary lesions ≥ 50% on CTA in the detection of significant coronary lesions on coronary angiography or the occurrence of clinical events

The sensitivity, specificity, positive predictive value, negative predictive value, and accuracy of a) a positive troponin result in the second measure; b) troponin variations of 20%; and c) coronary lesions ≥ 50% on CTA for detection of lesions ≥ 70% on coronary angiography or for prediction of clinical events at 30 days are presented in Table 2 . The overall performance of CTA for detecting the composed outcome was better than troponin measures.

**Table 2:** – Performance of the second measure of positive troponin, troponin variation of 20%, and coronary lesions ≥ 50% at computed tomography angiography in detecting lesions ≥ 70% at coronary angiography or to predict clinical events at 30 days

	Sensitivity	Specificity	Positive predictive value	Negative predictive value	Accuracy
Second positive troponin result	12.1%	88.1%%	33.3%	67.0%	63.0%
Troponin variation ≥ 20%	33.3%	73.1%	37.9%	69.0%	60.0%
Coronary lesions ≥ 50% at CTA	93.9%	89.6%	81.6%	96.8%	91.0%

*CTA: computed tomography angiography.*

## Agreement analysis in patients who underwent coronary angiography

### Agreement between sensitive troponin variations and the presence of significant coronary lesions on coronary angiography

This analysis included only patients who underwent coronary angiography (N=38). Using the presence of significant coronary lesions on coronary angiography as the gold standard, the Kappa agreement test with a positive troponin result in the second measure showed slight agreement (kappa = 0.006, p = 0.922). The best cut-off for troponin variation regarding the presence of significant coronary lesions on coronary angiography was 20%. Area under ROC curve for the 20% variation in sensitive troponin was 0.465 (CI 95%: 0.230 – 0.701) ( Figure 2 ).

### Performance of sensitive troponin measures in the detection of significant coronary lesions on coronary angiography

The sensitivity, specificity, positive predictive value, negative predictive value, and accuracy of a) a positive troponin result in the second measure, and b) troponin variations of 20% in the detection of lesions ≥ 70% at coronary angiography are presented in Table 3 .z Significant coronary lesions were detected by coronary angiography in 81.6% of patients with lesions ≥ 50% on CTA. This proportion was higher than the one for positive troponin in the second measure or its variations. High specificities were found for a positive troponin result in the second measure and troponin variations of 20% - 85.7% and 71.4%, respectively.

**Table 3:** – Performance of the second positive troponin result or troponin variation of 20% in detecting lesions ≥ 70% on coronary angiography

	Sensitivity	Specificity	Positive predictive value	Negative predictive value	Accuracy
Second measure of positive troponin	12.9%	85.7%%	80%	18.2%	26.3%
Troponin variation ≥ 20%	32.3%	71.4%	83.3%	19.2%	39.5%

## Discussion

Our study presents the performance of sensitive troponin measures or CTA in the detection of significant coronary lesions on coronary angiography and/or clinical events in patients with chest pain and intermediate risk for cardiovascular events. Sensitive troponin measures agreed poorly with the detection of significant coronary lesions on CTA or coronary angiography and with the occurrence of clinical events. CTA was superior to the measure of serial troponin, with better sensitivity and negative predictive value in the detection of coronary artery disease. In clinical practice, patients with acute chest pain are often discharged from the hospital according to chest pain protocols based only on the serial measurement of troponins. Our findings suggest that intermediate risk patients without ischemic alterations on the electrocardiogram should preferably be stratified at admission by CTA. This strategy may reduce the chance of erroneous hospital discharge in these situations. We emphasize the fact that the assessment of clinical events becomes secondary in this context due to the sample size, and hence the agreement with the diagnosis of coronary heart disease was the most relevant finding.

It is not uncommon that patients with chest pain are released from emergency rooms after initial evaluation and develop ischemic events in the following hours. These individuals do not receive an adequate treatment at the appropriate time.^3 , 9^ It is estimated that one in eight patients with unstable angina will suffer an acute myocardial infarction (AMI) in the next two weeks. Mortality in patients with AMI admitted or mistakenly released from the emergence departments ranges from 6% to 25%,^3^ which leads to lawsuits related to medical malpractice.^4^ The incidence of adverse outcomes in patients with TIMI risk score of 3 and 4 can reach up to 11.1%.^10 , 11^ However, studies exploring diagnostic strategies for chest pain evaluation in this specific population are scarce and come mostly from secondary analyses.

In 2006, Morris et al.^8^ conducted a study including 1,000 consecutive patients with acute chest pain to explore whether the use of TIMI risk score could help predict combined events at 30 days in this population. The AUC was 0.79 (CI 95%: 0.75 - 0.84), which showed a good applicability of the score. Since then, studies have suggested that patients with intermediate scores (3 and 4) should have troponin measured and a provocative ischemic test performed, when possible.^8 , 12^ Our findings also suggest that it is important to better evaluate the presence of coronary heart disease in this population using a non-invasive test in addition to troponin measures.

Using two serial measurements of sensitive troponin with short time intervals is a good approach to rule out acute coronary syndromes in low-risk patients, which allows the implementation of rapid chest pain assessment protocols.^5 , 6 , 13 , 14 - 31^ A study on patients with suspected ACS showed that a 20% increase in high-sensitivity troponin levels were associated with greater probability of ACS, with an area under the ROC curve of 0.785. Other studies observed that variations in high-sensitivity troponin T over a few hours also had high negative predictive values.^32^ However, it is worth noting that the population included in most studies that showed a very high accuracy of ultrasensitive troponin had a low risk. The negative predictive value of a 20% variation in sensitive troponin in our study was 69%, an index below what the literature has shown, which may be justified by the inclusion of patients at intermediate risk.

In ROMICAT study, the use of CTA in addition to the TIMI risk score increased the accuracy for event prediction.^33^ The ROMICAT-II study included 1000 patients with chest pain and first negative troponin result, who were randomized to perform CTA or follow the usual chest pain protocol.^34^ During the 2-year follow-up including 333 patients, it was observed that CTA had high predictive power with an AUC of 0.61 for combined cardiovascular events. When associated with the TIMI score, the AUC reached 0.84. In this study, only 5.4% of patients had TIMI risk between 3 and 4.^35 , 36^ We believe our findings add to these results as we showed that, in intermediate risk patients, CTA performed better than serial measures of sensitive troponin.

When compared to traditional chest pain protocols, CTA does not alter outcomes such as death or AMI, however it reduces length of hospital stay and the number of unnecessary hospital admissions.^5 , 7 , 37 - 56^ Litt et al.^7^ showed that the use of CTA, compared with the traditional chest pain evaluation protocol, had an excellent safety profile in low-risk patients, as no death or AMI occurred at 30 days. Additionally, CTA promoted a higher number of hospital discharges (49.6% vs. 22.7%) and shorter hospital stay (18 hours vs. 24.8 hours, *p* < 0.001). The agreement between CTA and cardiac catheterization findings also seems to be strong.^44 , 45^ In ROMICAT-II, hospital stay was 7.6 hours shorter in the CTA group compared to the usual care group. In our study, we also observed that the time interval between patient arrival and CTA was approximately one hour shorter than the time between patient arrival and the result of the second troponin.

Our results should be interpreted in light of some limitations. This was a single center study, with a relatively small sample size, and the number of clinical events was low. Therefore, larger studies should be performed to validate our findings. Intermediate risk patients were rarely represented in previous studies investigating strategies for chest pain evaluation. We believe our findings add to the existing literature and suggest that CTA may have an important role in ruling out acute coronary syndromes in this population.

## Conclusions

CTA performed better than sensitive troponin measures in the detection of significant coronary disease in patients with chest pain and intermediate risk for cardiovascular events.
